# Predicting virus mutations through statistical relational learning

**DOI:** 10.1186/1471-2105-15-309

**Published:** 2014-09-19

**Authors:** Elisa Cilia, Stefano Teso, Sergio Ammendola, Tom Lenaerts, Andrea Passerini

**Affiliations:** MLG, Département d’Informatique, Université Livre de Bruxelles, Boulevard du Thriomphe - CP 212, 1050 Brussels, Belgium; Interuniversity Institute of Bioinformatics in Brussels (IB)², ULB-VUB, La Plaine Campus, Triomflaan - CP 263, 1050 Brussels, Belgium; Department of Computer Science and Information Engineering, University of Trento, via Sommarive 5, I-38123 (Povo) Trento, Italy; Ambiotec sas, R&D, Via Appia Nord 47, 00142 Cisterna di Latina (LT), Italy; AI-lab, Vakgroep Computerwetenschappen, Vrije Universiteit Brussel, Pleinlaan 2, 1050 Brussels, Belgium

## Abstract

**Background:**

Viruses are typically characterized by high mutation rates, which allow them to quickly develop drug-resistant mutations. Mining relevant rules from mutation data can be extremely useful to understand the virus adaptation mechanism and to design drugs that effectively counter potentially resistant mutants.

**Results:**

We propose a simple statistical relational learning approach for mutant prediction where the input consists of mutation data with drug-resistance information, either as sets of mutations conferring resistance to a certain drug, or as sets of mutants with information on their susceptibility to the drug. The algorithm learns a set of relational rules characterizing drug-resistance and uses them to generate a set of potentially resistant mutants. Learning a weighted combination of rules allows to attach generated mutants with a resistance score as predicted by the statistical relational model and select only the highest scoring ones.

**Conclusions:**

Promising results were obtained in generating resistant mutations for both nucleoside and non-nucleoside HIV reverse transcriptase inhibitors. The approach can be generalized quite easily to learning mutants characterized by more complex rules correlating multiple mutations.

## Background

HIV is a pandemic cause of lethal pathologies in more than 33 million people. Its horizontal transmission trough mucosae is difficult to control and treat because the virus has a high virulence and it infects several type of immune surveillance cells, such as those characterized by CD4 receptor (CD4+ cells). The major problem in treating the human virus infection is the drug selectivity since the virus penetrates in the cell where it releases its genetic material to replicate itself by using the cell mechanisms. A drug target is the replicating apparatus of the cell. HIV antiviral molecules will be directed against several cells such as macrophages or lymphocytes T to interfere with viral replication. The HIV releases a single-strand RNA particle, a reverse transcriptase and an integrase into the cell cytoplasm. Quickly the RNA molecule is retro-transcribed in a DNA double strand molecule, which is integrated into the host genome. The integration events induce a cellular response, which begins with the transcription of the Tat gene by the RNA polymerase II. Tat is a well-known protein responsible for the HIV activation since it recruits some cytoplasm host proteins involved in the expression of viral genes. Remarkably, HIV can establish a life-long latent infection by suppressing its transcription, thus making ineffective the large part of antiviral drugs aimed at controlling the viral replication. However replicating viruses adopt several drug resistance strategies, for instance, HIV induces amino acid mutations reducing the efficacy of the pharmaceutical compounds. The present work is aimed at gaining knowledge on mutations that may occur into the viral RNA transcriptase [1]. This is an important target to develop antiretroviral medicines and different types of molecules have been found active: the Nucleoside Reverse Transcriptase Inhibitors (NRTI) and Non NRTI (NNRTI). Although RNA RT inhibitors are active, the HIV virus is capable of quickly changing the RNA RT encoding sequence thus acquiring drug resistance. The antiviral therapy is based on the use of cocktails of molecules including new RNA RT inhibitors. A computational approach to predict possible mutation sites and their sensibility to drug is thus an important tool in drug discovery for the antiretroviral therapy.

Computational methods can assist here by exploring the space of potential virus mutants, providing potential avenues for anticipatory drugs [2]. To achieve such a goal, one first needs to understand what kind of mutants may lead to resistance. A general engineering technique for building artificial mutants is referred to as *rational design* [3]. The technique consists in modifying existing proteins by site directed mutagenesis. It relies on a deep domain knowledge in order to identify candidate mutations that may affect protein structure or function. The process typically involves extensive trial-and-error experiments and is also aimed at improving the understanding mechanisms of a protein behavior.

In this work we report on our initial investigation to develop an artificial system mimicking the rational design process. We consider two increasingly complex learning settings and corresponding learning techniques. In the first one we rely on a training set made of single amino acid mutations known to confer resistance to a certain class of inhibitors (we will refer to this as mutation-based learning). An Inductive Logic Programming (ILP) learner [4] is trained for each class of inhibitors in order to extract general rules describing mutations conferring resistance to the drug class. The learned rules are then used to infer novel mutations which may induce similar resistance. In the second setting we learn directly from mutants (comprising of up to 51 amino acid mutations) that have been experimentally tested for their resistance to the same classes of inhibitors (we will refer to this as mutant-based learning). This second setting is actually the most common situation, in which one is presented with a number of mutants together with some evidence of their susceptibility to certain treatments, but no clear information on which mutation is responsible for their behaviour. In this setting we employ a statistical relational learning approach [5] capable of learning weighted combinations of relational rules discriminating between groups of instances, drug-resistant vs drug-susceptible mutants in our case. The learned model is then used to generate novel mutants together with a score indicating their predicted resistance.

Machine learning methods have been previously applied to mutation data for predicting the effects of non-synonymous single nucleotide polymorphisms on protein stability [6], function [7–11], and drug susceptibility [12]. All of the these predictors make use of pure statistical learning techniques (Bayesian classifiers [7, 8], neural networks [9], random forests [10], support vector machines [11]) in combination with a large variety of sequence, structural, and functional features. A recent evaluation of the predictive performance of mutation prediction methods can be found in the review by Thusberg *et al.* [13].

To the best of our knowledge, the present paper is the first attempt to learn relational features of mutations affecting protein behavior and use them for generating novel relevant mutations. Modeling mutant resistance with relational rules provides two key advantages. First, the learned rules can be easily interpreted by human experts, providing valuable insights into the mechanisms of drug resistance. Second, while previous work focused uniquely on the identification of resistance mutations, our method can natively produce novel candidate mutations that are likely to confer greater fitness/resistance to a drug.

In the case of single mutations, it is straightforward to generate a set of potentially resistant mutations simply by testing all candidates with any of the above predictors. The same procedure, however, does not scale to the multiple case, where exhaustive enumeration is infeasible. On the contrary, our method can be readily extended to produce mutants with two or more mutations: the learned rules effectively constraint the space of candidate mutants, drastically reducing the (exponential) number of candidates. Additionally, it is possible to augment our approach by employing a more sophisticated statistical predictor to further characterize the generated mutants. Although in the experimental evaluation of the present work we focus on single residue mutations, we are actively working on extending our approach to generate mutants characterized by multiple mutated residues.

We report an experimental evaluation focused on HIV RT. RT is a well-studied protein: a large number of mutants have been shown to resist to one or more drugs and databases exist that collect those data from different sources and make them available for further analyses [14]. We tested the ability of our approach to generate drug-resistant amino acid mutations for NRTI and NNRTI. Our results show statistically significant improvements for both drug classes over the baseline results obtained through a random generator. A preliminary version of this work was presented in [15].

The approach can be in general applied in mutation studies aimed at understanding protein function. By searching for residues most likely to have a functional role in an active site, the approach can for instance be used in the engineering of enzyme mutants with an improved activity for a certain substrate.

## Methods

### Datasets

We applied our approach to predict HIV RT mutations conferring resistance to two classes of inhibitors: NRTI and NNRTI. The two classes of inhibitors differ in the targeted sites and rely on quite different mechanisms [16, 17]. NNRTI inhibit the reverse transcriptase by binding to the enzyme active site, therefore directly interfering with the enzyme function. NRTI are instead incorporated into the newly synthesized viral DNA for preventing its elongation.

We compiled two datasets ={(*x*,*y*)∈×}, where is the input space where the examples *x* are drawn from, either the space of single mutations or the space of mutants, depending on the learning setting (respectively, mutation-based and mutant-based learning). Examples *x* are expressed in the form of ground facts as well as the labels *y*, which represent the targets of the prediction. For instance, ={resistant, non-resistant} (corresponding to {true, false}) in the mutation-based learning setting and with respect to a specific inhibitor.

The former (Dataset 1) is a dataset of amino acid mutations derived from the Los Alamos National Laboratories (LANL) HIV resistance database [18] by Richter et al. [19], who used it to mine relational rules among mutations. It consists of 95 amino acid mutations labeled as resistant to NRTI and 56 labeled as resistant to NNRTI, over a set of 581 observed mutations. For the mutant-based setting, we collected (Dataset 2) HIV RT mutation data from the Stanford University HIV Drug Resistance Database. The database provides a dataset of selected mutants of HIV RT with results of susceptibility studies to various drugs, and was previously employed [12] for predicting drug resistance of novel (given) mutants^a^. It is composed of 838 different mutants annotated with susceptibility levels (low, medium and high) to drugs belonging to the NRTI (639 mutants) and NNRTI (747 mutants) drug classes. We considered a setting aimed at identifying amino acid mutations conferring high susceptibility (with respect to medium or low), and considered a mutant as highly susceptible to a drug class if it was annotated as being highly susceptible to at least one drug from that class.

### Learning in first order logic

Our aim is to learn a first-order logic hypothesis for a target concept, i.e. mutation conferring resistance to a certain drug, and use it to infer novel mutations consistent with such hypothesis. We rely on definite clauses which are the basis of the Prolog programming language. A definite clause *c* is an expression of the form:


where h and the b_*i*_ are atomic literals. Atomic literals are expressions of the form p(t_1_, …, t_*n*_) where p/n is a predicate symbol of arity n and the t_*i*_ are terms, either constants (denoted by lower case) or variables (denoted by upper case) in our experiments. The atomic literal h is also called the head of the clause, typically the target predicate, and b_1_AND … AND b_*n*_ its body. Intuitively, a clause encodes the fact that the head will hold whenever the body holds. For instance, a simple hypothesis like:


indicates that a mutation C in the proximity of a binding site confers to mutant A resistance against a certain drug (nnrti).

A clause *c* is said to cover a mutant if the mutant is classified as resistant according to the clause, i.e. if the head is true. Learning in this setting consists of searching for a set of definite clauses *H*={*c*_*i*_,…,*c*_*m*_} covering all or most positive examples, and none or few negative ones if available. Standard ILP techniques, such as Aleph, Golem [20], Progol [21], and FOIL [22], employ one of two opposite strategies to search the space of hypotheses. In bottom-up learning, the search starts from the most specific clause (allowed by the language bias) that covers a given example, which is then generalized until it cannot be further generalized without covering any negative examples. Generalization of the current clause relies on applying a generalization operator, which either i) substitutes a variable to a constant, or ii) removes a literal from the body. Conversely, top-down approaches start from the true hypothesis, which entails all examples, and gradually specialize it to reduce its coverage of negative examples. Clause specialization is performed by i) substituting a constant to a variable, or ii) adding a literal to the body. While these strategies are (necessarily) heuristic, it is possible to control the complexity of the hypothesis space by choosing an appropriate language bias. A more detailed treatment of the learning algorithm used by Aleph can be found in the Algorithm overview section.

The learned first-order clauses can be interpreted as relational features that characterize the target concept. The main advantage of these logic-based approaches with respect to other machine learning techniques is the expressivity and interpretability of the learned models. Models can be readily interpreted by human experts and provide direct explanations for the predictions. On the other hand, purely logic-based approaches fail to incorporate uncertainty in the hypotheses they produce, and different degrees of importance of the clauses of which hypotheses are made. Statistical relational learning [23, 24] techniques aim at filling this gap by combining statistics and expressive representational languages in developing predictive models. A simple and effective solution consists of learning a weighted combination of clauses, where clauses and their weights are jointly learned in trying to model the concept of interest.

In the biological domain, ILP has been successfully applied to a variety of learning problems, such as predicting sequence-based homology and gene/protein function [25], finding regularities in microarray data [26], modeling protein–ligand [27] and protein–protein interactions [28], discovering pharmacophores [29], and drug design [30, 31].

### Background knowledge

We built a relational knowledge base for the problem domain. Table 1 summarizes the predicates that we included as a background knowledge. We represented the amino acids of the reference wild type (consensus sequence) with their positions in the primary sequence (position/2) and the specific mutations characterizing them (mut/4). Target predicates were encoded as resistance of the mutation or mutant to a certain drug (res_against/2).

**Table 1:** **Background knowledge predicates**

Background knowledge predicates	
position(AA,Pos)	Indicates an amino acid in the wild type sequence
mut(MutID,AA,Pos,AA1)	Indicates a mutation: mutation or mutant identifier, position and amino acids involved, before and after the substitution
res_against(MutID,Drug)	Indicates whether a mutation or mutant is resistant to a certain drug
color(Color,AA)	Indicates the coloring group of a natural amino acid
typeaa(T,AA)	Indicates the type (e.g. aliphafatic, charged, aromatic, polar) of a natural amino acid
same_color_type(AA1,AA2)	Indicates whether two amino acids belong to the same coloring group
same_typeaa(AA1,AA2,T)	Indicates whether two amino acids are of the same type T
same_color_type_mut(MutID, Pos)	Indicates a mutation to an amino acid of the same coloring group
different_color_type_mut(MutID, Pos)	Indicates a mutation changing the coloring group of the amino acid
same_type_mut_t(MutID, Pos, T)	Indicates a mutation to an amino acid of the same type T
different_type_mut_t(MutID, Pos)	Indicates a mutation changing the type of the amino acid
aamutations(Pos,AA1,AA2,Num)	Indicates whether a given mutation requires at least a single, double, or triple nucleotide substitution
close_to_site(Pos)	Indicates whether a specific position is close to a binding or active site if any
location(L,Pos)	Indicates in which fragment of the primary sequence the amino acid is located
conservation(Pos,ConsClass)	Indicates whether a position is highly conserved or not
in_ss(SS,N,Pos)	Indicates whether a mutation occurs within the Nth secondary structure element of a given type
in_motif(Pos,Motif)	Indicates whether a mutation occurs within a known sequence motif
catalytic_propensity(AA,CP)	Indicates whether an amino acid has a high, medium or low catalytic propensity
mutated_residue_cp(Rw,Pos,Rm,CPold,CPnew)	Indicates how, in a mutated position, the catalytic propensity has changed (e.g. from low to high)

Summary of the background knowledge facts and rules. MutID is a mutation or a mutant identifier depending on the type of the learning problem.

Note that this encoding considers mutations at the amino acid rather than nucleotide level, i.e. a single amino acid mutation can involve up to three nucleotide changes. Focusing on single nucleotide changes would have drastically expanded the space of possible mutations. We thus kept the focus on amino acid mutations but we included the cost (in terms of nucleotide changes) of a certain amino acid mutation to further refine our search procedure as explained in the following.

Additional background knowledge was included in order to highlight characteristics of amino acids and mutations. To this aim we devised all the subsequent predicates: typeaa/2 indicates the type of the natural amino acids according to the Venn diagram grouping based on the amino acids properties proposed in [32]. For example, a serine is a tiny and polar amino acid. color/2 indicates the type of the natural amino acids according to the coloring proposed in [33] and reported in Table 2. For example the magenta class includes basic amino acids as lysine and arginine while the blue class includes acidic amino acids as aspartic and glutamic acids. These groups of amino acids do not overlap as in the previous case. same_type_aa/3 indicates whether two amino acids belong to the same type T, i.e. a change from one amino acid to the other conserves the type of the amino acid. same_color_type/2indicates whether two amino acids belong to the same coloring group, i.e. a change from one amino acid to the other conserves the coloring group of the amino acid. same_type_mut_t/3indicates that an amino acid substitution at a certain position does not modify the amino acid type T with respect to the wild type. For example mutation i123v conserves the aliphatic amino acid type while mutation i123d does not (i.e. different_type_mut_t/3 holds for it). same_color_type_mut/2indicates that an amino acid substitution at a certain position does not modify the amino acid coloring group with respect to the wild type. For example mutation d123e conserves the blue amino acid group while mutation d123a does not (i.e. different_color_type_mut/2 holds for it). aamutations/4indicates whether a given amino acid mutation can be triggered by a single, double, or triple nucleotide substitution. For instance to change an alanine a into an aspartic acid d a single nucletotide substitution can be sufficient as in the case a: gct →d: gat.

**Table 2:** **Amino acid types encoded in color classes**

Color class	Amino acids	Description
Red	AVFPMILW	Small and/or hydrophobic and/or aromatic
Blue	DE	Acidic
Magenta	RK	Basic
Green	STYHCNGQ	Hydroxyl and/or polar and/or basic

Classification of amino acid types in color classes originally proposed in [33] and used to define the color/2predicate.

The predicates color/2, same_color_type/2, and same_color_type_mut/2 have been originally proposed in [34]. Other background knowledge facts and rules were devised in order to express structural relations along the primary sequence, secondary structure, and catalytic propensity of the involved amino acids: close_to_site/1 indicates whether a specific position is less than 5 positions away from a residue belonging to a binding or active site. In our specific case, the background theory incorporates knowledge about a metal binding site and a heterodimerization site. location/2indicates in which fragment of the primary sequence the amino acid is located. Locations are numbered from 0 by dividing the sequence into fragments of 10 amino acid length. conservation/2indicates whether a position is highly conserved or not. Conservation is defined in terms of positional variation (entropy) among a curated multiple-alignment of reverse transcriptase sequences, taken from the LANL HIV resistance database [35]. in_ss/3indicates whether a mutation occurs within a known secondary structure element. We encoded position specific knowledge for the four secondary structure classes: helix, strand, turn, and coil, through the predicates helix/1, strand/1, turn/1, and coil/1. This information was derived from the 3D model of the RT structure by using the DSSP program [36]. in_motif/2indicates whether a mutation occurs within a known sequence motif. Our background theory includes information about PROSITE [37] and Pfam motifs [38]. catalytic_propensity/2indicates whether an amino acid has a high, medium or low catalytic propensity according to [39]. mutated_residue_cp/5indicates how, in a mutated position, the catalytic propensity has changed (e.g. from low to high).

Conservation, secondary structure, and features encoding the closeness to the active site are among the standard features used by mutation effect predictors [8, 40]. Motifs have been used for a number of tasks, such as the identification of non-neutral single nucleotide polymorphisms [41].

### Algorithm overview

**Figure 1:**
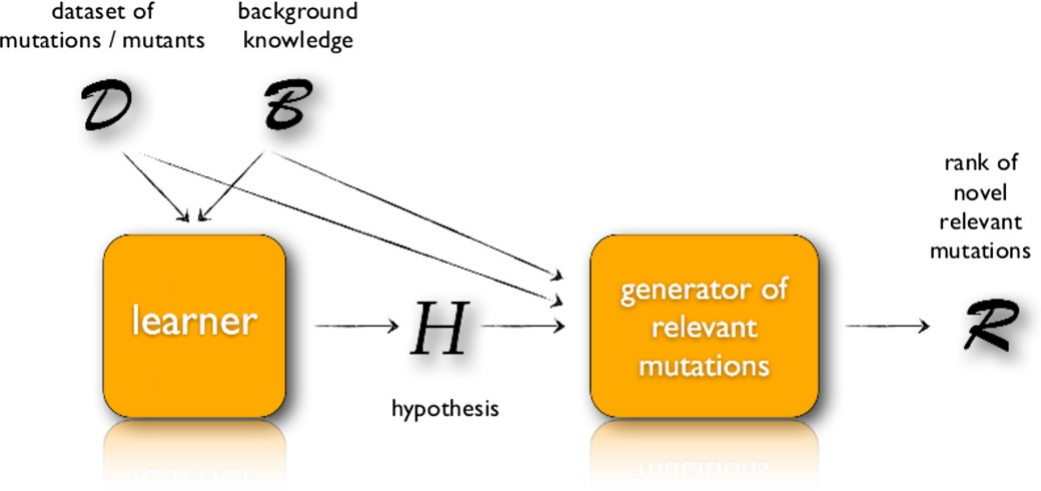
**Mutation engineering algorithm.** Schema of the mutation engineering algorithm.

The proposed approach is sketched in Figure 1.

#### Step 1: Learning phase

The first step is the learning phase. A learner is fed with a logical representation of the data and of the domain knowledge to be incorporated, and it returns a first-order logical hypothesis *H* for the concept of mutation conferring resistance to a certain class of inhibitors.

In this context there are two suitable ways to learn the target concept, depending on the type of input data and their labeling:

- the one-class classification setting, learning a model from positive instances only. This is the approach we employ for Dataset 1: positive examples are mutations for which experimental evidence is available that shows resistance to a drug, but no safe claim can be made on non-annotated mutations.
- the binary classification setting, learning to discriminate between positive and negative instances. This setting is appropriate for Dataset 2: positive examples are in our experiments mutants labeled as highly susceptible to the drug class, negative examples are those with medium or low susceptibility.

In the one-class classification case we employ the Aleph (A Learning Engine for Proposing Hypotheses) ILP system [42], which learns first order logic hypotheses in a bottom-up fashion. It incrementally builds a hypothesis trying to cover all positive examples. The hypothesis search is guided by a Bayesian evaluation function, described in [43], scoring candidate solutions according to an estimate of the Bayes’ posterior probability that allows to trade-off hypothesis size and generality. Aleph adds clauses to the hypothesis based on their coverage of training examples. Given a learned model, the first clauses are those covering most training examples and thus usually the most representative of the underlying concept.

**Figure 2:**
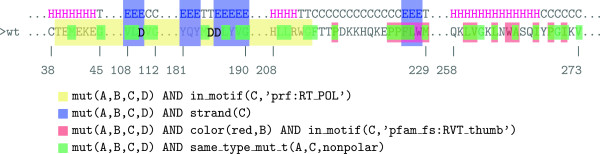
**Model for the resistance to NNRTI learned from Dataset 1.** An example of learned hypothesis for the NNRTI task with highlighted amino acid positions covered by the hypothesis clauses.

In Figure 2 we show a simple example of hypothesis covering a set of training mutations from Dataset 1. The learned hypothesis models the ability of a mutation to confer resistance to NNRTI and is composed of four first-order clauses, each one covering different sets of mutations of the wild type as highlighted in colors: yellow for the first clause, blue for the second, red for the third, and green for the fourth one. Some mutations are covered by more than one clause as shown by the color overlaps. For instance, a mutation of the glycine in position 190 satisfies three clauses: the first, the second and the fourth. On top of the RT consensus sequence we also report the corresponding secondary structure annotation, by highlighting in magenta the helices and in blue the *β*-strands. The PROSITE and Pfam motifs prf:RT_POL and pfam_fs:RVT_thumb appearing in the clauses identify specific regions along the RT sequence. Bold letters in the picture indicate residues involved in the RT metal binding site (D110, D185 and D186).

**Figure 3:**
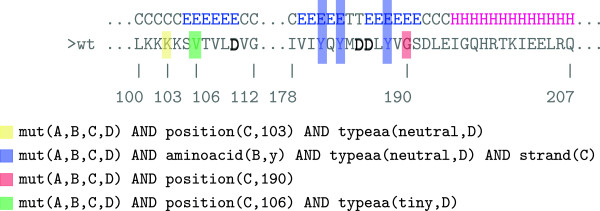
**Model for the resistance to NNRTI learned from Dataset 2.** An example of learned hypothesis for the NNRTI task with highlighted amino acid positions covered by the hypothesis clauses.

In the binary classification case, we employ kFOIL [5], a statistical relational approach which learns a weighted combination of clauses discriminating positive from negative instances. kFOIL is a kernel-based approach [44], capable of learning hypotheses made of complex non-linear combinations of clauses. For the sake of interpretability we limit ourselves to second degree polynomial kernels, where the predictive model is a combination of conjunctions of up to two clauses.In Figure 3 we show an example with a few clauses extracted from the hypothesis learned on one of the dataset partitions generated during the experimental evaluation (see Results section). As in the above example, the model is composed of four first-order clauses, each contributing to the characterization of NNRTI resistance mutations. Three of the four clauses specify positions 103, 106 and 190 directly as likely targets for resistance conferring mutations. The second clause, which is not position specific, represents mutations of thyrosines occurring within a strand, where the mutation is a non-charged amino acid. Note that two clauses with distinct position predicates cannot be simultaneously satisfied by the same mutation. Conjunctions of clauses will thus typically involve one position-specific clause and one or more position-aspecific ones, where the latter further detail the features that likely resistant mutations at that position are expected to exhibit.

#### Step 2: Generative phase

The second step of our approach is the generative phase, in which the learned hypothesis is employed to find novel mutations that can confer drug resistance to an RT mutant. A set of candidate mutations can be generated by using the Prolog inference engine starting from the rules in the learned model. The rules are actually constraints on the characteristics that a mutation of the wild type should have in order to confer resistance to a certain inhibitor, according to the learned hypothesis.

Algorithm ?? details the mutation generation procedure. We assume, for simplicity, to have a model *H* for a single drug class. The procedure works by querying the Prolog inference engine for all possible variable assignments that satisfy the hypothesis clauses, each representing a mutation by its position and the amino acid replacing the wild type residue. The set of mutations generated by the model is ranked according to a scoring function *S*_*H*_ before being returned by the algorithm. When using Aleph, we define *S*_*H*_ as the number of clauses in *H* that a candidate mutation *m* satisfies. When using kFOIL, *S*_*H*_ is the value of the weighted combination of the satisfied clauses. The latter case allows a much more refined scoring, as will be showed in the experimental evaluation.

Algorithm for novel relevant mutations discovery.


Consider the example model in Figure 2. Among the mutations generated using the model are all those changing the glycine in position 190 in a non polar amino acid: 190P, 190A, 190F, 190I, 190L, 190V, 190M. Here 190P indicates a change of the wild type amino acid at position 190 into a proline. Each of these mutations satisfies the first, the second and the fourth clause, receiving a score of three. Note that mutation 190A is part of the known NNRTI surveillance mutations (see [45]).As for the model in Figure 3, the position specific rules all identify known surveillance mutations: 103N, 103S, 106M, 106A, 190A, and 190S. Clause two affects position 181, a thyrosine occurring within a strand, and corresponds to surveillance mutations 181C, 181I, 181V.

## Results

### Learning from mutations

We first learn general rules characterizing known resistance mutations (from Dataset 1) to be used for predicting novel candidate ones.

We divided the dataset of mutations into a training and a test set (70/30) in a stratified way, which means by preserving, both in the train and test set, the proportion of examples belonging to one of the two drug classes. This produces a training set of 106 mutations and a test set of 45 ones.

We trained the ILP learner on the training set and we evaluated on the test set the set of mutations generated using the learned model. The evaluation procedure takes the set of generated mutations and checks which of them appears in the test set. We compare the recall of the approach, i.e. the fraction of test mutations generated by the model, with the recall of a baseline algorithm that generates a set (of the same cardinality) of random mutations. By random mutation we mean here the mutation at a random position in the wildtype into a randomly chosen amino acid, different from the one occurring in the wildtype at that position. A random generation is admittedly a rather simple baseline, but it is useful in highlighting the amount of reduction of the search space (the space of mutations) achieved by our algorithm. In order to fully exploit this gain in exploration efficiency, the algorithm should be extended to generate mutants with multiple mutations. This is the subject of our future work, as discussed in the Conclusions.

**Figure 4:**
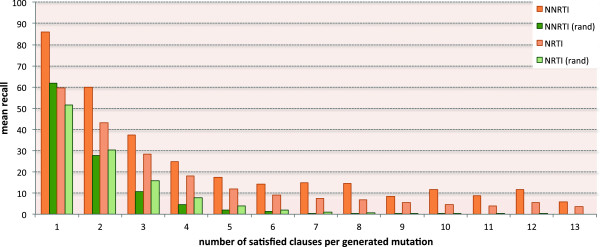
**Mean recall trend by number of satisfied clauses (Dataset 1).** Mean recall of the generated mutations on the resistance test set mutations from Dataset 1 by varying the number of satisfied clauses. The mean recall values in orange refer to the proposed generative algorithm. The mean recall values in green refer to a random generator of mutations.

We computed 30 random 70/30 train/test splits and performed 30 runs of our algorithm on each split (Aleph has a random component generating the seed for the hypothesis search). Figure 4 reports results averaged over all runs for both NNRTI and NRTI tasks. In this setting, the average size of the learned hypotheses for NNRTI and NRTI are 10 and 14 rules respectively. The figure shows the mean recall on the test set when increasing the score threshold for accepting a mutation, i.e. the number of clauses a mutation must satisfy in order to be accepted. The results of the random baseline consider the same number of mutations selected by the method for each threshold. The recall trend is shown in orange for our approach and in green for the random generator for both classes of inhibitors. Recall differences are statistically significant according to a paired Wilcoxon test (*α*= 0.01).

We finally learned a model on the whole dataset in order to generate a single set of mutations for further inspection. We report five examples of novel mutations with the highest score for each one of the tasks: S105Y, S105T, S105N, S105G, S105C for NNRTI and 50A, 63A, 63M, 159L, 195V for NRTI. For NNRTI, known resistance mutations are found in positions 103 and 106, possibly explaining the high score of mutations at position 105. In [46], the authors found a set of novel mutations conferring resistance to efavirenz and nevirapine, which are NNRTI. Our mutation generation algorithm partially confirms their findings. Apart from mutation 138Q, not generated by our model, all other mutations have been generated, with 90I satisfying two out of five clauses and 101H, 196R, and 28K satisfying one.

Table 3 reports the most commonly learned clauses for both NNRTI and NRTI classification tasks. The rules for NNRTI resistance give relevance to mutations in *β*-strands, while for NRTI, mutations on turns and coils seem to be more relevant. It is also evident that the most susceptible region for developing resistance to these inhibitors is the region between positions 54 and 234 along the primary sequence, corresponding to the motif prf:RT_POL. In addition, for the resistance to NNRTI the region between positions 98 and 107 is more relevant, while for NRTI it is the region between positions 64 and 71 (see the location predicate).

**Table 3:** **Most frequent learned clauses (Dataset 1)**

# models	Learned clause
**NNRTI**	
21.8	mut(A,B,C,D) AND strand(C)
20.5	mut(A,B,C,D) AND location(11,C)
17.1	mut(A,B,C,D) AND strand(C) AND in_motif(C,’prf:RT_POL’)
9.9	mut(A,B,C,D) AND in_motif(C,’pfam_fs:RVT_1’)
9.4	mut(A,B,C,D) AND same_type_mut_t(A,C,neutral) AND strand(C)
7.9	mut(A,B,C,D) AND color(red,D) AND in_motif(C,’prf:RT_POL’)
7.3	mut(A,B,C,D) AND same_type_mut_t(A,C,nonpolar)
6.8	mut(A,B,C,D) AND in_motif(C,’prf:RT_POL’)
6.1	mut(A,B,C,D) AND color(red,B)
5.9	mut(A,y,C,D)
**NRTI**	
25.2	mut(A,B,C,D) AND location(7,C)
18.8	mut(A,B,C,D) AND in_motif(C,’prf:RT_POL’)
16.1	mut(A,B,C,D) AND turn(C) AND in_motif(C,’prf:RT_POL’)
12.1	mut(A,B,C,D) AND same_type_mut_t(A,C,neutral) AND in_motif(C,’prf:RT_POL’)
11.3	mut(A,B,C,D) AND coil(C) AND conservation(C, high)
11.1	mut(A,B,C,D) AND conservation(C, high)
11	mut(A,B,C,D) AND same_color_type_mut(A,B) AND in_motif(B,’prf:RT_POL’)
8.7	mut(A,B,C,D) AND same_color_type_mut(A,B)
7.3	mut(A,B,C,D) AND in_motif(C,’pfam_fs:RVT_1’)
7.3	mut(A,B,C,D) AND color(red,B) AND in_motif(C,’prf:RT_POL’)

List of the ten most frequent rules learned on Dataset 1, sorted by average number of models they appear in.

### Learning from mutants

The next set of experiments is focused on learning mutations from mutant data (Dataset 2). Learned models are still limited to single amino acid mutations, and so are novel mutants generated by the system.

We randomly assigned the mutants in Dataset 2 to 30 train/test set splits, by avoiding having mutants containing the same resistance mutation (according to the labelling used in Dataset 1) in both training and test sets. For each of the 30 splits, we evaluated the recall of the generated mutations on the known resistance mutations (from Dataset 1), by first removing all the mutations that were also present in the training set. Comparison is again made on a baseline algorithm generating random mutations.

**Figure 5:**
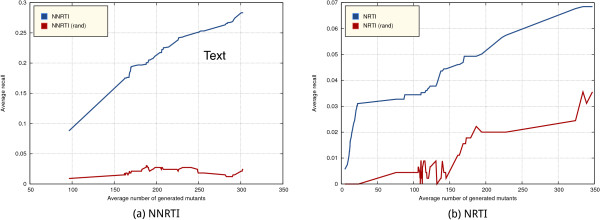
**Mean recall of the generated mutations on the resistance test set mutations from Dataset 2 by varying the threshold on the prediction confidence, and the corresponding average number of overall generated mutations (i.e., not necessarily in the test set), in blue.** The red line refers to the random generator of mutants. **(a)** Left panel: results for the NNRTI case. **(b)** Right panel: results for the NRTI case.

Results averaged on the 30 random splits are reported in Figure 5. The curve shows the average recall of the generated mutations while varying the threshold over their confidence, and the corresponding number of overall generated mutations. For NNRTI, we can see that we obtain an average recall of 25% while generating only 250 mutants, and can reach up to 27% with about 300 generated mutants. In both cases the results are statistically significantly higher than those achieved by a random generator (paired Wilcoxon test, *α*= 0.01).

The hypothesis for the resistance to NNRTI identifies more than half (12 out of 18) of the known resistance surveillance mutations reported in [45]: 103N, 103S, 106A, 181C, 181I, 181V, 188L, 188C, 190A, 190S, 190E, all with very high confidence. The model also predicts other not previously reported mutations as being resistant with high confidence, for instance 183F and 232A, very close to known surveillance mutations 181C and 230L.

Also in the case of NRTI the generative algorithm suggests most (32 of 34) known surveillance mutations reported in [45]: all of them except those targeting position 69 (including an insertion).

Table 3 lists the most frequently learned clauses in the 30 distinct models learned during the cross-validation procedure. It is easy to see that the most frequent clauses tend to favor mutations to positions 103, 106, and 143 for NNRTI resistance and 165 and 188 for NRTI resistance, among other less frequent positions. The clauses also specify properties of the mutations occurring at these positions. On the one hand, NNRTI resistant mutations are predicted to have a strong preference for strand residues (with strand occurring three times in Table 4) and for non-charged mutations. On the other hand, NRTI resistant mutations are predicted to occur within PROSITE motif RT_POL and Pfam motif RVT_1; mutations to highly conserved methionine positions are also predicted to confer resistance, as confirmed by surveillance mutation 184V.

**Table 4:** **Most frequent learned clauses (Dataset 2)**

# models	Learned clause
**NNRTI**	
All	mut(A,B,C,D) AND position(C,X)
9	mut(A,B,C,D) AND position(C,103) AND typeaa(neutral,D)
6	mut(A,B,C,D) AND position(C,106) AND typeaa(tiny,D)
6	mut(A,y,C,D) AND typeaa(neutral,D) AND strand(C)
6	mut(A,y,C,D) AND strand(C)
5	mut(A,B,C,a) AND position(C,106)
5	mut(A,y,C,D) AND typeaa(neutral,D)
4	mut(A,B,C,D) AND position(C,90) AND correlated_mut(A,C,E)
4	mut(A,B,C,D) AND position(C,143) AND same_type_aa(D,B,polar)
3	mut(A,B,C,D) AND typeaa(aromatic,B) AND strand(C) AND typeaa(neutral,D)
**NRTI**	
All	mut(A,B,C,D) AND position(C,X)
17	mut(A,m,C,D) AND same_type_aa(B,D,nonpolar)
13	mut(A,m,C,D) AND highconservation(C)
12	mut(A,w,C,D)
9	mut(A,m,C,D) AND inMotif(C,pfam_ls:RVT_1)
9	mut(A,m,C,D)
9	mut(A,p,C,D)
6	mut(A,B,C,D) AND position(C,165) AND correlated_mut(A,C,E)
6	mut(A,B,C,D) AND position(C,188) AND correlated_mut(A,C,E)
6	mut(A,m,C,D) AND inMotif(C,prf:RT_POL)
6	mut(A,m,C,D) AND inMotif(C,pfam_fs:RVT_1)

List of the ten most frequent learned rules for Dataset 2, sorted by number of models they appear in. The table also includes the clause position(C,X), which is present in all models for different values of X.

## Discussion and future work

The results shown in the previous section are a promising starting point to generalize our approach to more complex settings. We showed that the approach scales from few hundreds of mutations as learning examples to almost a thousand of complete mutants. Moreover the learned hypotheses significantly constrain the space of all possible single amino acid mutations to be considered, paving the way to the expansion of the method to multi-site mutant generation. This represents a clear advantage over alternative existing machine learning approaches, which would require the preliminary generation of all possible mutants for their evaluation. Restricting to RT mutants with two mutated amino acids, this would imply testing more than a hundred million candidate mutants. At the same time our statistical relational learning approach cannot attain the same accuracy levels of a sophisticated technique modelling for instance the three dimensional rearrangements of the resulting mutant. We plan to combine the respective advantages of the two approaches by using our statistical relational model as a pre-filtering stage, producing candidate mutants to be further analysed by complex modelling techniques and additional tools evaluating, for instance, a mutant stability. An additional direction to refine our predictions consists of jointly learning models of resistance to different drugs (e.g. NNRTI and NRTI), possibly further refining the joint models on a per-class basis. On a predictive (rather than generative) task, this was shown [34] to provide improvements over learning distinct per-drug models.

Our approach is not restricted to learning drug-resistance mutations in viruses. More generally, it can be applied to learn mutants having certain properties of interest, e.g. improved or more specific activity of an enzyme with respect to a substrate, in a full protein engineering fashion.

## Conclusions

In this work we proposed a simple statistical relational learning approach applicable to mutant prediction and protein engineering. The algorithm relies on a training set of mutation data annotated with drug resistance information, builds a relational model characterizing resistant mutations, and uses it to generate novel potentially resistant ones. Encouraging preliminary results on HIV RT data indicate a statistically significant enrichment in resistance conferring mutations among those generated by the system, on both mutation-based and mutant-based learning settings. Albeit preliminary, our results suggest that the proposed approach for learning mutations has a potential in guiding mutant engineering, as well as in predicting virus evolution in order to try and devise appropriate countermeasures. In the next future we plan to generalize the proposed approach to jointly generate sets of related mutations shifting the focus from the generation of single amino acid mutations to mutants with multiple mutations.

## Endnote

^a^**Genotype-Phenotype Datasets.**http://hivdb.stanford.edu/cgi-bin/GenoPhenoDS.cgi
